# Impairment in Working Memory and Executive Function Associated with Mercury Exposure in Indigenous Populations in Upper Amazonian Peru

**DOI:** 10.3390/ijerph191710989

**Published:** 2022-09-02

**Authors:** Alycia K. Silman, Raveena Chhabria, George W. Hafzalla, Leahanne Giffin, Kimberly Kucharski, Katherine Myers, Carlos Culquichicón, Stephanie Montero, Andres G. Lescano, Claudia M. Vega, Luis E. Fernandez, Miles R. Silman, Michael J. Kane, John W. Sanders

**Affiliations:** 1 Department of Psychology, Wake Forest University, Winston-Salem, NC 27109, USA; 2 Center for Energy, Environment, and Sustainability, Wake Forest University, Winston-Salem, NC 27109, USA; 3 Wake Forest School of Medicine, Winston-Salem, NC 27101, USA; 4 Emerge, Emerging Diseases and Climate Change Research Unit, School of Public Health and Administration, Universidad Peruana Cayetano Heredia (UPCH), San Martin de Porres 15102, Peru; 5 Centro de Innovación Científica Amazónica, Puerto Maldonado 17001, Peru; 6 Department of Biology, Wake Forest University, Winston Salem, NC 27109, USA; 7 Carnegie Amazon Mercury Project, Department of Global Ecology, Carnegie Institution for Science, 260 Panama Street, Stanford, CA 94305, USA; 8 Department of Psychology, University of North Carolina at Greensboro, Greensboro, NC 27412, USA

**Keywords:** methylmercury, working memory, executive functions, indigenous population, environmental exposure, Matsigenka, Manu National Park, Amazon Basin

## Abstract

The Matsigenka people living traditional lifestyles in remote areas of the Amazon rely on a fish-based diet that exposes them to methylmercury (MeHg) at levels that have been associated with decreased IQ scores. In this study, the association between Hg levels and working memory was explored using the framework of the Multicomponent Model. Working memory tasks were modified to fit the culture and language of the Matsigenka when needed and included measures for verbal storage (Word Span) visuospatial storage (Corsi Block Task) and a measure of executive functions, the Self-Ordered Pointing Task (SOPT). An innovation of the Trail Making Tests A & B (TMT A & B) was pilot tested as another potential measure of executive functions. The mean hair Hg levels of 30 participants, ages 12 to 55 years, from three different communities (Maizal, Cacaotal and Yomibato) was 7.0 ppm (sd = 2.40), well above the World Health Organization (WHO) limit for hair of 2.0 ppm and ranged from 1.8 to 14.2 ppm, with 98% of a broader sample of 152 individuals exceeding the WHO limit. Hair Hg levels showed significant associations with cognitive performance, but the degree varied in magnitude according to the type of task. Hg levels were negatively associated with executive functioning performance (SOPT errors), while Hg levels and years of education predicted visuospatial performance (Corsi Block accuracy). Education was the only predictor of Word Span accuracy. The results show that Hg exposure is negatively associated with working memory performance when there is an increased reliance on executive functioning. Based on our findings and the review of the experimental research, we suggest that the SOPT and the Corsi Block have the potential to be alternatives to general intelligence tests when studying remote groups with extensive cultural differences.

## 1. Introduction

Mercury (Hg) is a heavy metal and neurotoxin that is damaging to humans and wildlife and is a persistent global environmental pollutant that can be emitted from both natural and anthropogenic sources [1]. As an element, it is a contaminant that can be exceptionally long-lived, taking tens of thousands of years to be cleared from landscapes by moving into fauna and flora, being exported to other ecosystems, or becoming permanently buried [2]. The largest contributor of mercury to the environment is artisanal and small-scale gold mining (ASGM), which accounts for over 37% of the global total of mercury emitted from all sources [3].

All chemical forms of Hg are toxic to humans. Exposure to several forms can cause deleterious effects on the central and peripheral nervous systems, cardiovascular system, urinary system, immune system, skin, and lungs [4,5]. Of most concern is the organic and highly bioavailable form, methylmercury (MeHg) [6,7]. Primarily produced in aquatic environments through the methylation of elemental mercury by microorganisms, MeHg is a potent neurotoxin that readily accumulates in living organisms and biomagnifies within food webs, becoming enriched in high trophic levels of freshwater and marine ecosystems [8,9,10]. Humans with diets that consume such higher trophic level organisms, such as Amazonian indigenous populations [11] are at elevated risk of methylmercury exposure through diet [12].

The Matsigenka in the upper Madre de Dios watershed in Southeastern Peru is one group of people susceptible to Hg poisoning because of their reliance on fish as a main source of protein [11,13,14]. Estimated to number around 1000, the Matsigenka families living inside the protected areas of Manu National Park (MNP) are geographically restricted and culturally traditional relative to the lives and practices of Matsigenka in nearby regions [15]. They maintain a lifestyle that includes the practice of swidden agriculture, hunting with bow and arrow, fishing, and gathering [13,14] with the bulk of their animal protein coming from migratory fish known to have high concentrations of mercury [16]. The Peruvian government maintains primary schools in all villages and health posts in the most inhabited communities but living conditions inside the park are restricted to protect the ecosystems of MNP and the Matsigenka culture and include prohibitions on the types of everyday objects and practices that one would readily find in the nearest towns outside of the park [17]. The improvement of education, health, nutrition, and basic services (drinkable water and solar electricity) has led to migration and integration of groups of Matsigenka living in isolation in the headwaters of the river. There are other groups in voluntary isolation that are neither contacted nor registered, but their number is unknown.

Studies with Amazonian peoples show that high levels of Hg are associated with decreased cognitive functioning. Impairments are typically assessed with Intelligence or IQ tests that are a battery of standardized measures summarized into a composite score and normalized against a known population. Recent work has showed that for Peruvian children each one unit increase in log hair mercury levels was associated with a 2.59-point decrease in the IQ index for General Cognitive Ability [18]. Similarly, studies of children in the Amazon region of Brazil reported that for every 10 ppm Hg increase there was a decrease of half a standard deviation in estimated IQ scores [19]. These dose-related effects have also been reported outside of Amazonia regions, such as the Faroe Islands where longitudinal work has found that every 10-ppm increase in Hg is associated with a 1.8 to 2.2-point mean decrease in IQ score [20]. The findings are consistent with other studies conducted across the globe [21,22,23] and provide strong evidence (with exceptions reported from studies in the Seychelle Islands [23,24] and the United States [25] that exposure to MeHg negatively impacts the general intelligence of humans.

Despite their breadth and validity, standardized intelligence tests have long been suspected of educational, cultural, and language biases [26] and present unique challenges when being used with indigenous people. Discerning how impairment is related to elemental toxins becomes difficult or impossible when used to assess people whose environment and lifestyle does not match the origin of the test, and when the regionally validated norms used for evaluation force participants to use a second language and counting system [27]. 

These challenges are particularly striking when considering the culturally isolated and in some cases recently contacted Amazonian indigenous populations. For example, the forward and backwards digit span task [28], included in many intelligence batteries or as a measure of short-term memory [19], requires the recall of digits from a base-10 number system. Some Amazonian indigenous groups use a “one, two, many” counting system that does not include discrete indicators for items of three or above [29,30]. Remote communities vary greatly in how much formal schooling is available, leading to differences in instruction in Spanish and the use of Arabic numerals. When IQ tests are administered in a second language and with a less familiar set of knowledge, the true scores of participants may be obscured and could exaggerate deficits due to environmental toxins. The concern has not gone unnoticed [31,32] and are supported by studies that report differences between urban Amazonian groups and rural indigenous people on intelligence tests [18,33].

One way to minimize cultural bias in cognitive testing is to shift away from using broad standardized test batteries and instead focus on select cognitive processes that are well-understood in contemporary theory and can be measured with tools tailored for a specific population. To this end, the construct of working memory is promising for a closer examination of how mercury may affect intelligence and cognitive functioning, and previous Hg investigations with Amazonian children that have measured working memory using sub-scales of IQ tests have found them to be sensitive to levels of hair Hg levels [18,19]. Working memory has been conceptualized in many ways [34,35,36] from a domain-free model based on time [37] to one based on attention resources [38]. (For a comparison among models, see [34,35,36]. One of the earliest and most productive models is the Baddeley Multicomponent Model that presents working memory as a network of interacting components including short-term memory storage for different types of information (verbal and visual-spatial) to be held temporarily or rehearsed. Operating concurrently with the storage components is a “central executive” that is used when tasks demand attention and cognitive control beyond passive storage and rehearsal [39,40,41].

The experimental work from the Multicomponent Model and others has led to results suggesting that working memory is key to understanding the fluid aspects of intelligence that are assessed in standardized IQ tests [42]. Kane et al. ([43], p. 170) describe working memory span tasks as measures that “tap a very general—and very important—cognitive primitive” that contributes to individual scores in general intelligence factors. More recently, Shipstead et al. ([44], p. 773) argued that “…working memory capacity and fluid intelligence arise from similar cognitive mechanisms but are reliant on these mechanisms to different degrees”. In support of this view, correlations between measures of complex working memory tasks and measures of general fluid intelligence or “Gf” are less than perfect, but robust (r = 0.59, [45]; r = 0.65, [45]). Although not equivalent to IQ scores [46], select working memory measures that involve the use of executive functions [47] may provide coarse estimates of associations between Hg and the cognitive processes underlying the IQ score. Considering these relationships among assessment tools could provide an effective workaround to measure cognitive impairments when there are extensive cultural differences among people living in areas of with Hg exposures.

In this study, we first sought to measure hair Hg levels of Matsigenka residents living in three villages within the restricted area of Manu National Park in Madre de Dios, Peru. Testing inside MNP has been limited given the isolation and restrictions imposed in the area, but assessment is important for a full understanding of how MeHg is reaching communities living in the headwaters far from, but still connected by river and migratory fish to, ASGM activity. Second, we sought to understand how exposure to MeHg may impact human cognitive functions by focusing on the construct of working memory in lieu of traditional IQ tests. Three tasks were used to measure working memory in this study, one for each component of storage and executive functions: verbal short-term memory (Word Span), visuospatial short-term memory (Corsi Block) and central executive processing (Self-Ordered Pointing Task or SOPT). These tasks were chosen because they have a strong tradition within working memory research and the stimuli and instructions can be easily adapted for the Matsigenka as needed. Additionally, the tasks allow for a comparison of performance across components that employ central executive processes to varying degrees. ‘Simple’ storage measures like the verbal word span should not rely on executive functions [47] and therefore, performance should not be associated with Hg levels. In contrast, a ‘complex’ working memory measure, like the SOPT, requires executive functions [47] and performance is expected to show associations with Hg levels in a similar way as an IQ test. Visuospatial storage tasks, such as the Corsi Block, are designed to capture capacity of the visual and spatial domain, but there is ample evidence that they elicit executive functions to similar extents as the more ‘complex’ working memory tasks [48,49,50]; thus, performance is also expected to be associated with Hg levels.

A fourth measure in this study is a pilot test for a new version of the Trail Making Test A & B (TMT-A, TMT-B). The TMT A & B is a clinical assessment of executive functions that is sensitive to a variety of brain injuries [51]. The version created for the Matsigenka communities replaces alphabet and numerical stimuli with non-verbal stimuli and alters the instructions to match cultural norms, but still demands the use of executive functions in the form of set shifting [52]. As a pilot test, the results from the TMT are considered separately from the other measures, but the same predictions regarding executive processes still apply and decreased performance is expected to be associated with higher levels of Hg levels.

The objective for this study is to learn about the levels of MeHg exposure for people who are living in the headwaters of the Manu River located inside the restricted zone of MNP and to investigate how that exposure is associated with impairment of cognitive functions. The cognitive measures were chosen or designed specifically for the Matsigenka residents. This study is novel because our approach forgoes the traditional IQ test and focuses singly on the construct of working memory using findings from the cognitive experimental literature for prediction. We highlight the relationship between central executive functions of working memory and those underlying intelligence testing. This cross-discipline work is important because it offers a strategy for studying how exposure to Hg is linked to cognitive impairment even when investigating people who live in isolated areas with cultures and languages that are distinct enough to render an IQ score uninterpretable [53].

## 2. Materials and Methods

**Figure 1:**
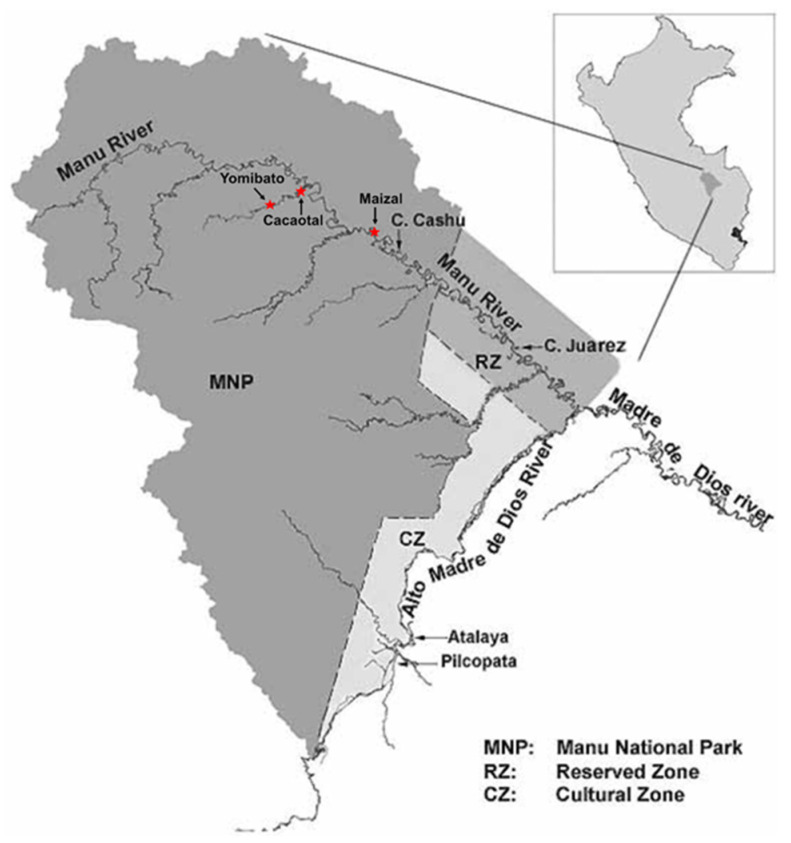
Map of Manu National Park, Peru. Location of Cacaotal, Yomibato, and Maizal are indicated by red stars on map.

Study site and population. Manu National Park (MNP) is Earth’s highest biodiversity park, consisting of 1.7 M ha of forested landscapes in the tropical Andes and adjacent Amazonian lowlands in SE Peru. Participants in this study come from three Matsigenka communities living along the Manu River inside the MNP: Maizal, Cacaotal and Yomibato, (Figure 1). All three communities are located within the upper Madre de Dios watershed, and collectively are situated about 180 Km away from any urban areas. Two cohorts of data collection are presented in this study. The first data collection was in June 2017 and was only from the community of Maizal. This collection was intended to test levels of Hg in hair samples from residents and to test the feasibility of the modified SOPT task with a Matsigenka sample. A total of 38 individuals, ages ranging from age 1 to 65 years, were given physicals and samples of blood and hair were collected. Twelve adults, ages between 22 and 65 years, were also administered the SOPT task in an abbreviated form to learn if the task had potential as a tool for assessing executive function. Two participants in the Maizal community in 2017 did not know their age but were determined during the physical exam and interview to be over 12 years old.

The second data collection was in June 2018 and included the Maizal community as well as the Cacaotal and Yomibato communities. A total of 114 individuals across the three communities were given a physical and neurological examination (Mini Mental Status Exam and Cranial Nerve Exam), and samples of blood and hair were collected. Participants who were ages 12 years or older and who provided a full set of responses to the Word Span task, Corsi Block task, and SOPT (n = 30). Three individuals from the Cacaotal community did not know their age or birthday but were judged to be at least 12 years old based on measures of size, maturation, and appearance.

For the pilot test of the TMT, the sample size is smaller. The TMT was given to a subset of 19 participants who were able to successfully complete the practice trials. The eligible individuals were from the villages of Cacaotal and Yomibato; none of the residents from the village of Maizal were able to complete the practice trials. A comparison of mean Hg hair levels conducted between the group included in the TMT sample (n = 19, mean hair Hg level = 6.43 ppm (sd = 3.80)) and those excluded from the TMT sample (n = 11, mean hair Hg level = 4.63 ppm (sd = 2.92)) did not show that there was a significant difference between groups, t = −1.45, *p* = 0.07.

For the remainder of this paper, the descriptions of protocol, data presented in the Results section, and interpretations come from the second cohort of data collection in 2018 from the residents of Maizal, Caocatal and Yomibato unless explicitly stated to be from the Maizal 2017 cohort or to include participants from both data collections.

Consent Process. Written informed consent was obtained from participants ages 18 or older with signature and/or fingerprint (in case of illiteracy) and for those under the age of 18 years, written consent was obtained from the parent or accompanying adult family member, as well as an informed assent for participants ages 12 or older. Ethics approval to conduct research on human subjects was granted through Wake Forest University Medical School Institutional Review Board (Human Subjects: IRB000044673) and the Institutional Ethics Committee of the Universidad Peruana Cayetano Heredia, Lima, Peru (N° 100806). The authorization to conduct the study in 2017 and 2018 within the Manu National Park was provided by the Peruvian National Protected Areas Service (Servicio Nacional de Áreas Naturales Protegidas por el Estado, or SERNANP by its Spanish acronym).

Mercury Assessment. Hair samples of approximately 0.5 g were collected from each participant. Hair was cut close to the occipital area of the scalp with stainless steel scissors, placed in paper envelopes inside of zip lock plastic bags with silica, and stored at room temperature. Hair samples were analyzed for total mercury (THg) using EPA Method 7473 (Mercury in Solids and Solutions by Thermal Decomposition, Amalgamation, and Atomic Absorption Spectrophotometry; EPA 1998) on a Milestone DMA-80 dual-cell Direct Mercury Analyzer at the Mercury and Environmental Chemistry Laboratory (LAMQA) laboratory in Puerto Maldonado, Madre de Dios, Peru. Exposure to THg was used as a proxy of exposure to MeHg as more than 90% of Hg in hair is MeHg [54].

Physical Exam and Neurological Assessment. Central nervous system assessment included measures of head circumference and a comprehensive cranial nerve exam. Peripheral nervous system assessment involved tests for abnormalities in reflexes, strength, sensation, tremor, and gingivitis. Motor systems dysfunction was screened for by testing balance, bilateral coordination, upper extremity coordination, visual motor control, visuospatial organization, and upper extremity speed. During the examination, participants’ height was measured using a stadiometer and their weight was assessed using an electronic digital scale to calculate the body mass index. They were also interviewed about their education levels, family status, and diet and nutrition. From these assessments, two participants were determined ineligible for participation in the cognitive tasks due to potential mental disability and visual abnormalities. Another two participants were determined ineligible because they were not residents MNP but were only visiting temporarily.

Fish Consumption. During the interview, participants were asked, (1) “How frequently do you eat fish?” with responses ranging from “every day” to “every 5 days”, “never” or “other” as well as (2) “How many times per day do you eat fish generally?” with responses ranging from “1 time per day” to 5 times per day”, “never” or “other”. These two responses were used to create an index of total weekly fish consumption by dividing days in a week by the reported frequency (question 1), multiplied by the reported daily intake (question 2).

Blood tests. About 10 uL of blood samples from finger-prick were collected. Then, hemoglobin was analyzed in a portable photometric device; HemoCue^®^ Hb 201 DM system (HemoCue^®^ AB, Ängelholm, Sweden), which allowed it to measure 0–25.6 g/dL. Assessment of anemia was considered as <13.5 g/dL for males and <12 g/dL for females.

Cognitive assessments. Measures for each component of working memory are described below along with any adaptations made from the original, or most commonly used, measure. The cognitive tasks were administered in fixed order (as presented). All instructions and verbal stimuli were provided in the Matsigenka language and were administered through a native Matsigenka translator who traveled with the research team to translate Matsigenka into Spanish for test administration.

Word Span Task. Verbal short-term memory is commonly measured in intelligence batteries by presenting a short list of stimuli, such as numbers (as in the Digit Span task) or words (as in the Word Span task) and asking participants to recall the stimuli immediately after presentation. The Word Span is used in some intelligence batteries instead of Digit Span, particularly those designed for children such as the Woodcock-Johnson test [55]. For Matsigenka participants, the stimuli were lists of high-frequency, unrelated words as stimuli presented in the Matsigenka language. Words were chosen from three noun categories that would have multiple exemplars found within the environment and interactions of the daily lives of the Matsigenka living in MNP: Nature (14 items), Human (10 items), Functional Object (12 items). The list of thirty-six words were translated into both Spanish and Matsigenka and were determined by bilingual Matsigenka translators to be of regular frequency use in the everyday lexicon of the communities sampled (list presented in Supplemental Section S1). Word Span tests were administered in four set sizes (3, 4, 5, and 6 words), with two trials of each presented in ascending order. The test administrator read the words aloud at the rate of approximately one word per second. The participant was asked to orally recall the items in order. An accuracy score was calculated with full credit for recall of words in the correct order and half credit for words recalled out of order per trial. Scores per trial were summed and divided by the set size and then averaged across trials per participant.

**Figure 2:**
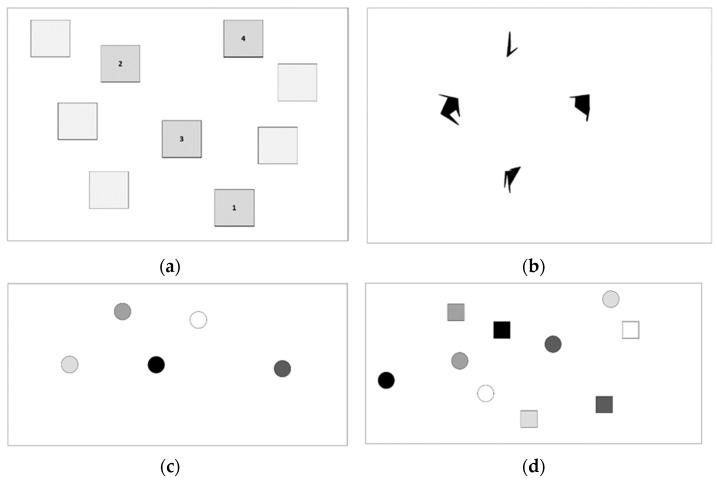
(**a**–**d**) Example Stimuli for Cognitive Tasks: (**a**) Example of Corsi Block set size 4. Numbers in square are used to demonstrate a sequential pattern but were not displayed to the participant. (**b**) Example of SOPT set size 4 with 4 different “Attneave” shapes, (**c**) Example of TMT A “Shades” presented in random locations, (**d**) Example of TMT B “Shades & Shapes” presented in random locations.

Corsi Block Task. The original Corsi Block Task was developed to assess hemispheric specialization between verbal and spatial processing [56,57] but variations have since been created for clinical diagnosis, experimental research, and intelligence testing. A traditional paper form of the Corsi Block Task was chosen here for its longstanding use across testing situations [58]. A single paper for presentation of locations was used across all trials. Stimuli were nine two-dimensional squares printed on paper in an asymmetrical pattern (see Figure 2a). The test administrator tapped a sequence of squares and participants immediately responded by tapping the same squares in order. Each participant’s responses were recorded by the test administrator on a separate response form. One practice trial of three squares was given before test trials began. If a participant responded with error, the same practice trial was repeated until the participant responded correctly. Test trials were of four different set sizes presented in ascending order (3, 4, 5, and 6), with two trials of each for a total of eight trials. An accuracy score was calculated with full credit for recall of locations in the correct order and half credit for locations recalled out of order per trial. Scores per trial were summed and divided by the set size and then averaged across trials per participant.

Self-Ordered Pointing Task (SOPT). The SOPT was originally developed to look for associations between frontal lobe functioning and working memory [51] and is now considered a well validated measure of central executive processes [59]. For Matsigenka living in remote areas of the Amazon with highly variable schooling, the advantages of the SOPT include that the test is free of language and number stimuli, can be administered without the use of a computer, and speeded response times are not required. The last two issues, administration without need for a computer and speeded response, are preferred testing formats given the context of the Matsigenka lifestyle and environment which does not involve computers or timed behaviors (for discussion on cultural influences in cognitive tasks including speed, see [60]).

During the SOPT, participants were presented with a set size of either 4, 6, or 8 shapes on a single sheet of paper (see Figure 2b for example). Stimuli in this version of the task was abstract Attneave shapes [61]. The use of shapes that are abstract is a departure from other versions that have been used in cultural adaptations of the task [62] but were chosen for two reasons: (1) to avoid participants’ use of a verbal strategy that may reduce the use of executive functions, and (2) to choose stimuli that could potentially be used with other groups of people being studied because of Hg exposure. During the practice and test trials, the same Attneave shapes were presented on each page during a trial but with the locations of each shape shifted between pages. Locations of shapes were random within an area on the page. As the test administrator turned pages, participants were to point to a new item on each page (i.e., an item not yet pointed to on that trial), but traditional instructions telling participants to point to a different location each time was not given to participants. (After data collection, response forms were checked for this strategy but there were no participants that pointed to the same location repeatedly.) Two trials of the set size of three were presented as practice prior to beginning test trials. Set sizes were given in ascending order with two trials per each set-size of 4, 6, and 8 items with errors totaled across all trials. The total number of errors was summed across set sizes and trials for the dependent variable, rather than accuracy in keeping with the scoring method used in the cognitive literature.

This version of the SOPT was piloted in 2017 with a small sample of adults in the Maizal community (N = 12). For this data collection, only the highest set size successfully completed set size, or “span” score was recorded. All 12 Maizal participants were able to correctly respond to at least one trial at the lowest level (set 4). Furthermore, included in that version was an additional set size of 10 shapes per page, but this level was determined to be too difficult and was removed from testing for the 2018 data collection.

Trail Making Test. Another well-regarded executive task, the TMT [63] is commonly used in clinical assessments of executive functions (also referred to as “cognitive flexibility” [64,65]. The TMT was first published as part of the Army Individual Test Battery in 1944 and was later adopted for research and diagnosis of hemispheric and frontal lobe dysfunction [66]. In the original TMT, participants are asked to connect numbers in ascending order (Version A or TMT-A) and then to alternate between connecting numbers and letters in alphabetical/numerical order (Version B or TMT-B) and the time to complete each trial is recorded. Time to complete (in seconds) can then be calculated as a difference score 64or ratio [65] between the B and A forms. For the Matsigenka, a novel version of the TMT was created to avoid the use of an alphabet or numbers. For Trail Making Test A “Shades” (Figure 2c), shaded circles replaced letters so that participants were asked to connect circles using a pen on a response sheet, ordering from lightest to darkest. For Trail Making Test B “Shades and Shapes” (Figure 2d), numbers were replaced with shapes. Participants were asked to alternate between connecting shades and shapes. For example, in Figure 2d, the participant would start at the white circle, move to the white square, then the lightest gray circle, then the lightest gray square until complete with the black circle and black square. The alternating between shapes does not match the original B version with regard to complexity because the number stimuli require participants to advance to different numerals in ascending order and the shapes remain constant between connections. It is possible that this could have decreased the difficulty of the “Shades and Shapes” version in comparison to the original TMT B with letters and numbers.

After two practice trials with three shades/shapes each, the test trials began with five trials of TMT A “Shades” followed by five trials of TMT B “Shades and Shapes”. Trials were scored as being successfully completed if all connections between shades and/or shapes were accurate. Typically, the TMT is a speeded task and cut-offs for deficiencies and impairments are determined by the number of seconds an individual requires to perform the task perfectly. For our versions, the subjects were instructed to emphasize accuracy and there was no mention of completing the task as quickly as possible.

Other versions of the TMT have been created that are language-free and use accuracy for the outcome measure but are still not appropriate for the Matsigenka because the modifications involve the use of numbers and/or involve memorizing stimuli [67]. Although using accuracy as a dependent variable eliminates the possibility of comparing outcomes with other studies using a traditional TMT task, it does not seem to change the underlying cognitive processes being captured, as demonstrated by a culturally adapted version of the TMT developed to assess Northern Aboriginal people in Northern Australia [62].

### Data Analysis

Descriptive statistics of all demographics, health, diet, and hair Hg levels and cognitive task variables were summarized across and between each community. Community comparisons with post hoc analysis and effect size calculations were calculated for most variables including cognitive tasks and Hg levels. For the TMT task, a *t*-test was done on the Hg levels between participants qualifying for the test trials and those ineligible for the test trials. Correlations and confidence intervals were calculated between all variables within the sample size of 30 participants and again separately for the sample of 19 participants who took the TMT tasks as part of the pilot test for the two versions. Because the three main predictor variables of Hg levels, Age, and Education were correlated, Variance Inflation Factors were calculated for each before running regression analyses. The missing ages for participants were replaced with the average age for the relevant community. The association between hair mercury levels and cognitive processing for three working memory tasks: Word Span, Corsi Block, and SOPT were tested using Ordinary Least Squares multiple linear regression. For each outcome, the same model was run using the covariates of Hg levels, Years of Education, and Age. All analyses were performed in JMP 16.0.0 (SAS Institute, Inc., Cary, NC, USA) for Windows software.

## 3. Results

### 3.1. Demographics and Health Indicators

The mean and frequency outcomes for each community are presented in Table 1 along with the summary statistics for the total sample across communities.

There were no physical or neurological impairments detected in any of the 30 participants included in the sample of cognitive measures, but some health indicators did suggest malnutrition and anemia. The mean BMI was 22.23 (sd = 3.46), with eight participants being underweight (BMI < 20) and the mean hemoglobin level was 11.81 (sd = 1.42), with 22 participants presenting anemia (females < 12 g/dL; males < 13.5 g/dL). Anemia is a variable of interest because it can be used as a proxy for chronic malnutrition [68,69,70], which is associated with poorer performance on intelligence measures [71]. Tests between communities did not reveal differences for BMI, *F* (2, 27) = 0.45, *p* = 0.64, ω^2^ = 0, or Hemoglobin, *F* (2, 27) = 1.5, *p* = 0.234, ω^2^ = 0.03. The calculated index of Fish Consumption ‘Total per Week’ did not show significant differences between communities, *F* (2, 27) = 0.29, *p* = 0.75, ω^2^ = 0. Education levels varied, with some participants from each community reporting no schooling (27%) and most participants reported either having attended some primary school education (40%) or some secondary school (33%). When counted in years of schooling from 0 to 12, the mean was 4.77 years (sd = 3.8). The community of Maizal reported the lowest mean years in education (1.33 years) followed by Cacaotal (4.82) and the highest mean years of education were in Yomibato (5.38). One participant in Yomibato reported having finished secondary school and was now serving as a teacher for the community. The comparisons between communities for years of education were not significant, *F* (2, 27) = 1.45, *p* = 0.25, ω^2^ = 0.03.

### 3.2. Hair Mercury Levels

**Figure 3:**
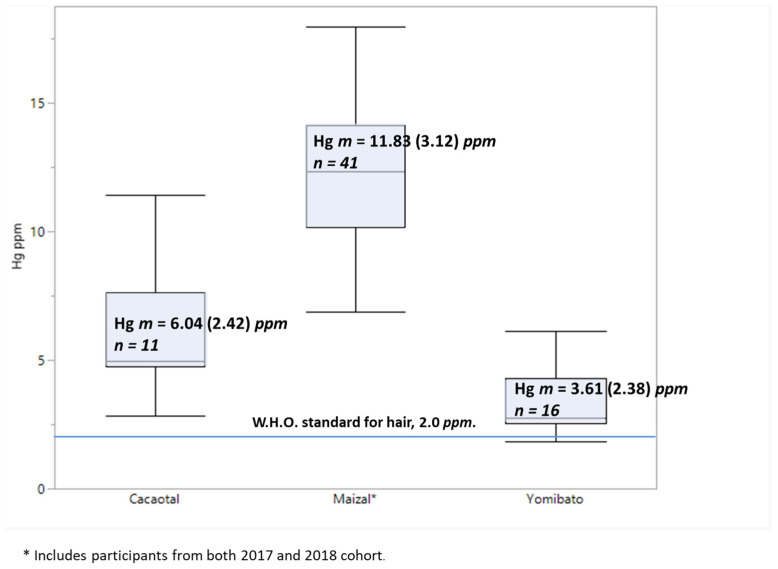
Hg levels per village with both the 2017 and 2018 cohort for the village of Maizal. Boxes show mean, interquartile range, and 2.5–97.5 percentiles.

Each participant’s Hg level was the average of two samples of hair strand analyses except for eight participants whose Hg level was based on the analysis from a single hair sample. Hg levels were high, with the mean Hg level across communities of 7.05 ppm, exceeding the WHO [72,73] recommended limits by 3.5×, with 98% of a broader sample of 152 individuals (across 2017 and 2018 data collections) exceeding the WHO limit, having mean Hg levels above the World Health Organization threshold limit of 2.0 μg/g. The exceptions were three individuals from Yomibato, who had levels of 1.81, 1.92, and 1.99 μg/g. An ANOVA test showed that Hg levels between groups were significant, *F* (2, 27) = 14.44, *p* < 0.01, ω^2^ = 0.47. Maizal residents had the highest mean Hg levels. Both collection years (2017 and 2018) found that Hg levels approached six times that of the threshold level [1,2]. The 2018 collection from Maizal had mean hair Hg levels of 11.49 ppm and the 2017 collection from Maizal (n = 38) was Hg level of 11.9 ppm (sd = 3.19, min = 2.4 ppm, max = 16.5 ppm). Figure 3. shows the observed hair Hg levels per village and includes the 2017 and 2018 data points for Maizal.

### 3.3. Cognitive Tasks

#### 3.3.1. Word Span

Across the three villages, the mean accuracy of words recalled was 0.52 (sd = 0.19). At the lowest set size of three items the mean accuracy was 0.87 (sd = 0.25) and 80% of participants were able to recall the words perfectly on at least one trial beyond the practice exercises. (Comparisons between set sizes for Word Span and subsequent tasks are presented in Supplemental Section S2). Differences in Word Span accuracy between communities were significant, *F* (2, 27) = 5.60, *p* < 0.01, ω^2^ = 0.23, as observed between Yombiato and Maizal (*p* = 0.018, Tukey’s HSD) but were not significance between Yomibato and Cacaotal (*p* = 0.07; Tukey’s HSD).

#### 3.3.2. Corsi Block

Across communities, the mean accuracy for recall of block locations was 0.59 (sd = 0.27). Comparisons of mean Corsi Block accuracy between communities was significant, *F* (2, 27) = 8.67, *p* < 0.01, ω^2^ = 0.34, and post hoc tests confirmed differences between Yombiato and Maizal (*p* < 0.01, Tukey’s HSD) and between Yomibato and Cacaotal (*p* = 0.01, Tukey’s HSD).

#### 3.3.3. Self-Ordered Pointing Test

The SOPT was scored by summing the number of errors across trials and set sizes rather than the accuracy (as was done with the Word Span and Corsi Block). The mean error score was 4.23 (sd = 1.65). At the lowest level, the mean error score was 0.47 (sd = 0.63) and 60% participants were able to complete the trial without any errors. Comparisons of mean SOPT errors between communities were not significant, *F* (2, 27) = 1.43, *p* = 0.25, ω^2^ = 0.03.

#### 3.3.4. Trail Making Task A and B

The sample for the TMT analyses was the participants who were age 12 or older, were able to complete the Word Span task, Corsi Block Task, and SOPT, and who also completed the practice trials for both the TMT A and TMT B task. Using these criteria for inclusion, the sample size decreased from 30 to 19 participants all from the communities of Cacaotal and Yomibato (see Table 2 for summary statistics and TMT scores for this sample). In the traditional version of the TMT task, the primary dependent variable is time (in seconds) to complete the task and a difference score is calculated between the time for Part A and time for Part B with the resulting score capturing the time to complete the “task switching” required in Part B [74]. This version of the TMT record accuracy instead of time. Difference scores for accuracy were not calculated because the accuracy on Part A “Shades” and Part B “Shades and Shapes” were correlated, *r* = 0.48, *p* = 0.03, and would be unreliable as a measure of performance. Instead, accuracy was considered separately for each part of the TMT and for the relationship to other variables such as Hg levels.

TMT A “Shades”. Mean proportion of correct responses across all trials and villages was 0.68 (sd = 0.35). Two participants were not able to answer any of the test trials correctly despite being able to complete the prior practice trials. Across all five trials, 42% of participants responded perfectly. Differences between the two communities, Yomibato and Cacaotal, were not significant, *t*(17) = 0.03, *p* = 0.48, d = 0.05.

TMT B “Shades & Shapes”. Mean proportion of correct responses across all trials and villages was 0.31 (sd = 0.32). Six participants were not able to answer any of the test trials correctly after successful completion of the practice trials. Only one participant completed all five trials of Part B with perfect accuracy. Performance between the two communities, Yomibato and Cacaotal, were significantly different, *t*(17) = 3.05, *p* < 0.01, d = 0.72 and paired *t*-test between TMT A and TMT B responses confirmed the difference between the two versions, *t*(18) = 8.56, *p* < 0.01, d = 1.17.

### 3.4. Correlations among Demographic Variables, Health, and Diet Indicators

The correlation matrix for all variables is presented in Table 3 and significant results are listed below along with 95% confidence intervals (CI). Hair Hg levels were positively correlated with Age, *r* = 0.50, [CI 0.17, 0.73], *p* < 0.01, but did not show a correlation with measures of Hemoglobin, *r* = −0.13, [CI −0.47, 0.24], *p* = 0.48, Body Mass Index, *r* = 0.11, [CI −0.26, 0.45], *p* = 0.57, or Fish Consumption as measured by the index Total per Week, *r* = −0.13, [CI −0.47, 0.24], *p* = 0.48. Education was negatively associated with Age, *r* = −0.47, [CI −0.71, −0.13], *p* < 0.01, indicating that the younger members of each community have more years of schooling than the older members. The measures of Fish Consumption, BMI, or Hemoglobin were not associated with any variables.

### 3.5. Correlations among Demographic Variables and Cognitive Tasks

Word Span accuracy was negatively related to Age, *r* = −0.56, [CI −0.76, −0.25] *p* = 0.01, and positively related to Education, *r* = 0.63, [CI 0.35, 0.81] *p* < 0.01. Corsi Block accuracy was also negatively related to Age, *r* = −0.44, [−0.69, −0.09] *p* = 0.01, and positively to Education, *r* = 0.59, [0.30, 0.79] *p* < 0.01. The correlation between errors on the SOPT and Education approached significance, *r* = −0.33, [CI = −0.62, 0.32] *p* = 0.07. Finally, Word Span accuracy and the Corsi Block accuracy were related, *r* = 0.62, [CI 0.33, 0.80] *p* < 0.01. The associations with Age and Education are not surprising given that declines in working memory with increased age are well-established [75] and the influence of education on intelligence tests with mercury exposed samples is also documented [18].

### 3.6. Correlations between Cognitive Tasks and Hair Hg Levels

Pearson’s correlations between each of the cognitive tasks and Hg levels can be found in Table 3. Hair Hg levels showed a significant negative relationship with Words Span accuracy, *r* = −0.38 [CI −0.65, −0.03], *p* = 0.04, as well as Corsi Block accuracy, *r* = −0.56 [CI −0.76, −0.25], *p* < 0.01 indicating that increased Hg levels are associated with poorer performance. The correlation between errors on the SOPT and Hair Hg levels was a significant positive, *r* = 0.41 [0.06, 0.67], *p* = 0.03, indicating that Hg levels increased so too did errors (i.e., poorer performance on the SOPT).

A separate correlation analysis was conducted for the sample of participants who participated in the TMT A and B versions and found that accuracy in both versions had a negative relationship to Hair Hg levels, but neither were significant, (TMT B “Shades and Shapes”, *r* = −0.35, [CI −0.69, 0.12] *p* = 0.14, and TMT A “Shades”, *r* = −0.36, [CI −0.70, 0.11] *p* = 0.13. Accuracy on the TMT A and B did correlate with each other, *r* = 0.48, [CI 0.03, 0.77], *p* = 0.04, but there were no other significant correlations between either TMT versions and the other demographic variables (age, education) or the health indicators (BMI, Hemoglobin). The Version B of the TMT showed significant correlations with the Corsi Block accuracy, *r* = 0.60, [CI 0.20, 0.83] *p* < 0.01 and a negative relationship with SOPT errors, *r* = −0.55, [CI −0.80, −0.13] *p* = 0.01 but, not with Word Span Accuracy, *r* = 0.31, [CI −0.16, 0.67], *p* = 0.20.

To understand how specific health and demographic factors may contribute to the observed relationships between Hg levels and the working memory components, a multiple regression analysis was conducted for each of the three cognitive outcome tasks (Word Span, Corsi Block, and SOPT). The models were limited to three predictor variables because of the small sample size across communities; the same three predictors were used for each of the cognitive tasks to allow for comparisons of fit and contribution. The first predictor, hair Hg levels, was chosen to test the hypothesis that Hg levels are associated with the components of working memory to different degrees, depending on the involvement of executive functions. The next predictors, Education and Age, were prioritized based on the strength and significance of their correlations with Hg levels. Although the three predictors were correlated, Variance Inflation Factors (VIF) were low, showing that multicollinearity was not an issue: Age (VIF = 1.63), Education (VIF = 1.29), Hg level (VIF = 1.33). Variables not included in the model were the health indicators of Fish Consumption, BMI, and Hemoglobin because they showed no significant correlation to other variables. Before running the model, the three participants in the Cacaotal community whose ages were unknown, were interpolated using the group mean age of 28 years. A regression analysis was not conducted on the TMT A and B data because of the smaller sample size and because the data collection was considered a pilot test of the measure.

The multiple linear regression results for each of the three cognitive tasks are presented in Table 4. For the Word Span Task, the model using Age, Education, and Hg levels as predictors was able to explain 50% of the variance in accuracy, but only one variable, Education, was a significant predictor.

**Figure 4:**
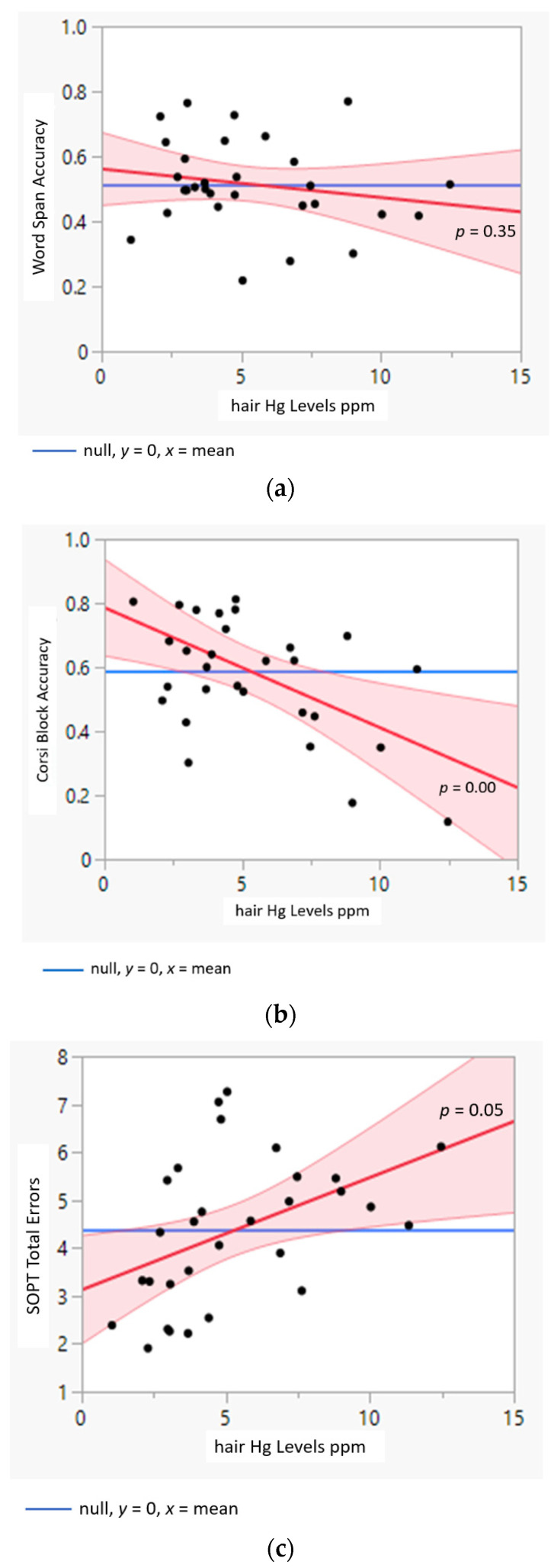
Added variable plots [76] showing the unique effect of a cognitive task (red line and associated 95% CI) as compared to a model assuming the model contains all the other terms (blue line) for each cognitive task but no relationship between the dependent and independent variable. (**a**) Word Span Accuracy vs. Hg, (**b**) Corsi Block Accuracy vs. Hg, (**c**) SOPT Total Errors vs Hg.

The same model predicting Corsi Block accuracy was able to explain 55% of the observed variance. Education and Hg levels, but not age, were significant predictors of Corsi Block accuracy. The model for SOPT errors were less predictive than for the other two cognitive tasks with 29% of variance explained. Hg levels significantly predicted errors on the SOPT when controlling for Age and Education. The predictor of Education approached significance when controlling for Age and Hg levels. The predictor of Age was not significant when controlling for Education and Hg levels. Importantly, responses on the Corsi Block and SOPT showed a continuous and quantitative response to Hg level across the range measured but was not significant for Word Span (Figure 4a–c).

## 4. Discussion

The levels of methylmercury in hair of thirty participants from three different indigenous communities along the Manu River were found to be above the maximum threshold of 2.0 ppm in all but three individuals, with levels exceeding the WHO limit [72,73] by an average of 3.5×. The results point to widespread elevated Hg levels in even the most remote indigenous populations living in watersheds with illegal artisanal and small-scale gold mining—in this case between 300 and 400 km upstream from the nearest mine. This study also explored the association between methylmercury exposure and working memory using cognitive tasks chosen to measure the short-term storage and executive functions. Previous studies exploring the impact of Hg exposure have mostly relied on IQ tests or working memory sub-scales that are likely to be influenced by culture, education, and language and would not be appropriate for the Matsigenka living inside the restricted area of MNP [18,19]. Here, some tasks were modified by changing stimuli, instructions, and outcome variables to make the tasks more consistent with traditional Matsigenka culture. Results suggest that Hg exposure may impair cognitive processes that rely on executive functioning, and that these effects are seen at even relatively lower levels of Hg exposure and increase monotonically with increasing Hg concentrations in hair. The findings highlight the risk to Amazonian indigenous populations, especially those living in areas impacted by ASGM where mining activity increases levels of Hg in fish that are consumed as a main part of the indigenous diet [77]. Below, we discuss the results within the framework of the Multicomponent Model, cultural considerations, their relevance to work using IQ tests, and how these tasks may be used in future studies and contribute to the Hg assessment field more broadly.

### 4.1. Hg Levels

Outcomes from the Hg assays establish that the mercury occurring in the environment exists at detectable and high concentrations in the Matsigenka residents of MNP. Across two collection years and the broad sample of hair Hg levels, 98% percent of the participants in this study showed levels of Hg hair above the 2.0 ppm threshold [ref]. For the second collection year, hair Hg levels were different between the three villages: Yomibato had the lowest mean levels (3.61 ppm), Cacaotal was almost double (6.04 ppm), and in the village of Maizal measures were almost doubled again (11.49 ppm). The relatively lower Hg levels in the residents of Yomibato and Cacaotal are consistent with levels reported by other studies of Amazonian people living outside of the park [9] while the elevated levels in the Maizal community are consistent with the range found in the 2017 data collection, which averaged just under 12 ppm with a larger sample (n = 41). The communities did not differ on fish consumption nor was the diet indicator (Total per Week) correlated with hair Hg level. This finding is not surprising because our measure of fish consumption did not account for the type or size of fish eaten. The trophic status of fish and where it is caught is known to be related to the level of Hg passed on to humans via fish consumption [8,9]. Each of the three communities fish in the part of the river that is nearest and most accessible to them, which may also influence outcomes. For instance, the community of Maizal catch fish in the main portion of the river where it may be easier to catch larger predatory fish such as catfish. Yomibato and Cacaotal are located on a small tributary that gives them access to different and smaller fish. A closer analysis of the diet of each of the communities is needed before the role of fish consumption in Hg levels can be determined.

### 4.2. Hg Levels and Working Memory Tasks

The association between Hg exposure and cognition was examined for separate theoretical components of working memory and the measures for each were based on well-established assessment tools that have been validated in experimental and clinical research but were modified specifically for the Matsigenka. Importantly, for prediction and hypothesis testing, the tasks also involved differing degrees of central executive functions.

#### 4.2.1. Verbal Short-Term Memory and Cultural Considerations

The word span task was used to measure verbal short-term memory and all participants demonstrated understanding and competency on practice trials and in the smallest set size, demonstrating participants’ ability to hold information temporarily. Recall declined as set size increased, demonstrating that the task was challenging enough to avoid ceiling effects in performance. Zero-order correlation between hair Hg levels and Word Span accuracy was moderate and significant, yet when fit to the regression model for Word Span that controlled for Education and Age, Hg level was no longer a significant predictor variable (see Figure 4a). At first glance, this may appear to be evidence for the null, that Hg has no effect on working memory; however, the finding is consistent with the Multicomponent Model and the hypothesis that Hg levels are associated with compromised central executive functions rather than the storage and recall of words [41].

Despite the simplicity of the word span task, consideration of the measure reveals the nuances of measuring cognitive abilities across diverse cultures. For instance, changes in how the results are scored and evaluated might determine if a score is interpreted as below “normal” or impaired. Many clinical assessments of short-term memory capacity, use an outcome that is reported as a “span” score or the average number of items that a person can temporarily hold and then recall (for discussion on span vs. accuracy calculations, see [78]. The average span score is reliably reported to be around seven for short-term memory [79] and four for working memory [80].

While it may seem straightforward to calculate the average span size among the sampled communities and compare it to these well-known metrics, that approach could be misleading when applied to indigenous groups like the Matsigenka. There are multiple known influences from language and culture that can introduce variation into a span score. One well documented is the Word Length Effect (WLE) that is the fluctuation in span size across languages depending upon the amount of time it takes to pronounce or rehearse words or numbers in a sequence [81,82,83]. For the Matsigenka, the WLE might occur if a participant’s span size in the Matsigenka language is different than their span size when speaking in a second language, Spanish. Span differences due to the WLE would not reflect an impaired ability to store and recall, but rather articulation time for the words in the sequence due to the structure of the language and the participant’s fluency.

Another cultural factor that could contribute to misleading differences when using a clinical norm to assess impairment is the counting systems of Amazonian groups. The traditional Matsigenka counting system categorizes items above three or four into a group of “many”. It is possible that the strategy of categorizing multiple items with the same descriptor of “many” indicates that a different rehearsal strategy is used when temporarily storing information [79,80]. The explanation has not been tested, but seems reasonable since anthropological writings about the Matsigenka have provided descriptions of individuals “losing count” of discreet number of objects when in a large amount, such as the account of a Matsigenka child over the age of six listing their possessions:

*If he says he has three needles, he has three. He begins to lose count only as numbers mount above five; like all his neighbors, he tends to remember in increments of five or ten and he can indicate these increments by opening his fists and flashing his fingers the right number of times.*

([84]. Families of the Forest, 2003, p. 153)

In a similar way, a culture’s system for categorizing information for memory could influence the internal processes of storing and retaining verbal-numerical items in memory. That is, the Matsigenka may or may not rely on articulatory rehearsal strategy, which is the repeating of words/digits between the presentation of the next stimuli and recall. If so, they could have decreased span size due to strategy, but not necessarily due to capacity.

The word span task used in this study was intended to measure verbal short-term storage of the Matsigenka in their own language and without comparison to a control-group norm, but the results cannot inform about potential word length effects, the influence of a different counting system on storage capacity, or a use of a strategy that is not articulatory rehearsal. None of these possibilities can be addressed with the current data but could influence the overall storage capacity estimates.

Although the word span task allows for illustrations of cultural diversity in psychological measurement, it can also be noted that none of these factors should have influenced the between-community differences that the current study found in Word Span accuracy scores and would only apply to those studies using normative data from outside the test group’s culture. The finding of differences between the communities as reported here are due to the factors measured (i.e., Hg levels or education) or others not yet explored. Further the pattern is consistent with other studies testing verbal short-term memory in a simple and complex format [19].

#### 4.2.2. Visuospatial Short-Term Memory

A negative relationship between Hg and the Corsi Block Task accuracy showed that the measure was sensitive to varied levels of Hg exposure. Higher hair Hg levels were associated with poorer visuospatial retention and recall, and this association remained significant after controlling for Age and Education (Figure 4c). However, this result does not necessarily mean that the storage capacity of visuospatial items is impaired exclusively. Although the task is often considered a measure of short-term storage in clinical assessment and as conceptualized in the Multicomponent Model, cognitive research provides ample evidence that the executive-processing contributions is higher than verbal span tasks, and on par with some domain-general tasks that are marked as measures of executive functions [48]. The association with Hg levels may be due to the impairment to the executive processes that are recruited during a visuospatial task and are the same that underlie the executive functions captured by IQ scores.

The evidence of Hg’s association with poorer visuospatial performance is partly consistent with other studies of Amazonian peoples. Santos-Lima et al.’s work (2020) with Amazonian children in Brazil found relationships between Hg hair levels and performance on the Corsi Block when using the backward recall version but not with the forward version. The order of recall (forward or backward) is thought to increase a task’s complexity by requiring the participant to reverse the sequence during recall and thus, causing the central executive to be more engaged [85]. While it is subjectively true that a backward task feels more difficult to participants, it may not be due to central executive contributions, as demonstrated in factor analysis studies on types of working memory tasks [42]. One possible reason for the difference in findings is that the children in previous Amazonian studies were living in more populated areas and recruited through their local schools. The fact that schools could organize recruitment for samples of children may indicate that there is greater stability, resources, and quality of formal education that can influence a child’s development of test scores or even teach memory strategy that is advantageous for such tests. With the current data, conclusion about the effects of school on task performance, but Education does seem to explain variance in cognitive scores in a way that is unique from Hg levels, as shown by the linear regression outcomes.

In the Faroe Islands, a cohort of residents was tested with a similar measure of visuospatial short-term memory, the Spatial Span, but did not find evidence that the Hg levels measured at birth negatively impacted recall in early adolescents [86] or young adulthood [20]. Instead, Spatial Span scores at age 14 years showed an unexpected positive correlation with Hg cord blood samples. It is not clear why there are disparate outcomes for correlations between visuospatial memory and Hg levels across studies, especially those found in opposite directions. Some reasons to consider are the scoring system used for the task, types of mercury assays during development (cord blood at birth vs. current hair levels), range of Hg detected within the sample, and larger sample sizes. Following up with more studies to understand how Corsi Block performance relates to hair Hg levels is worth exploring and could help to bridge our understanding of how visuospatial processing should be considered during assessment. For the Matsigenka studied in the current sample, poorer performance on the Corsi Block was associated with hair Hg levels and could be an important tool for assessing cognitive functions, providing an alternative path to capturing estimates of general cognitive impairment for Hg studies in ASGM active areas.

#### 4.2.3. Executive Functions

The major hypothesis offered in the cognitive literature and tested here, was supported by the results from the central executive task, the SOPT. The SOPT error score was positively correlated to Hg levels, so that participants with higher Hg levels were also more likely to make errors when remembering the previously selected shape. The association remained significant when Age and Education were controlled in a regression model (see Figure 4c). The SOPT task, though simple in form and instruction, has not been previously used in Hg studies but is often used in lab studies exploring models of working memory and intelligence. Similar to the Corsi Block, these findings in combination with the experimental cognitive literature showing that the SOPT correlates with intelligence tests [59], suggest that the SOPT could be a suitable substitute when intelligence tests are not appropriate for use in remote areas with indigenous people.

Previous studies with general working memory indices and executive tasks are consistent with the SOPT results. Studies with children in Amazonian Peru [18] and Brazil [19] found that working memory sub-scales included in IQ tests were correlated with Hg levels after controlling for covariates like socioeconomic measures and education. The finding that education is a common predictor across studies and even for each cognitive task in this study is interesting and highlights the influence of formal schooling on cognitive assessments (although it is also possible that cognitive abilities measured by our assessments might instead influence educational attainment). Just like the Word Span and Corsi Block tasks in this study, Education was correlated with SOPT error scores (*p* = 0.05) when Hg and Age were controlled. The measure of Education was years in school (up to 12th grade), which included a full range of scores (0 to 12 years), but it is unlikely that this metric captured the type or quality of education available in each of the communities. Based on our observations, there appeared to be disparate levels of educational resources among communities. While one village, Yomibato, had a building designated as classroom space for lessons; Cacaotal did not have classrooms, nor did they seem to have the basic supplies that would be expected in a classroom environment. Yomibato was also the only community with a drinkable water source close to the school. These resource differences are not reflected in participants’ reports of years in school. In other words, school attendance up to 4th grade in Cacaotal should not be assumed to be equal to attendance up to 4th grade in Yomibato. To account for differences in quality of education, it would be helpful to have additional indicators of community resources and organization.

Finally, this study presents data from a pilot test for a version of The Trail Making Test specifically created for the Matsigenka inside MNP as a measure of executive functions. There was evidence in the pilot test sample that the TMT “Shapes and Shades” was measuring executive functions: TMT B “Shapes and Shades” was significantly related to Corsi Block accuracy and the SOPT errors, but not to Word Span accuracy. Although the TMT B “Shades and Shapes” seems promising as a measure of executive functions, it is not certain that it could be used for detecting Hg associated impairment because the negative relationship between hair Hg levels and TMT B accuracy was not significant, r = −0.35, *p* = 0.12. Other issues for the TMT A & B pilot were that the tasks proved difficult for participants across communities; some could not successfully complete the practice trial and only one participant successfully finished both Parts A & B without error.

It could be that the shaded stimuli chosen for the TMT tasks were difficult to distinguish between, making the task visually challenging and also caused the two versions (A and B) to become conflated. Forms A & B are intended to capture different cognitive processes [87] with the simple version (A) replacing letters for shades, and the complex version (B) replacing numbers with shapes (see Figure 2c,d). The TMT B “Shades and Shapes” did require participants to alternate between decision sets, executive function. However, the results of both Part A and Part B in this study were correlated, which suggests that the underlying processes were similar or overlapping. This may be due to fact that the second dimension in Part B, the shapes, did not change incrementally as the numbers do in the original version. Overall, the TMT “Shapes and Shades” should be further investigated as an assessment tool, ideally in a larger sample and with more varied stimuli.

### 4.3. Limitations and Future Directions

A limitation of this work was the modest number of participants with fully completed cognitive tasks and health assessments. The numbers reflect the challenges of collecting data from remote regions. Even so, the three villages considered for this project were chosen for their location within the most remote areas of the protected zone of the park and at different points along the Manu River. Each community visited could be described as small communities with some degree of illiteracy present in each. These factors make assessment of cognitive impairment difficult and associations with Hg could possibly be obscured by other factors. Despite the sample size we had reasonable power (70%) to detect correlations as small as r = 0.40. The sample size may have also led to the sample not being representative of the Matsigenka population living inside the park, although this is difficult to know for certain. There are records of communities, especially the younger residents who have attended school, but the ages and other demographics of adults are sometimes unknown, and it is difficult to determine how many people are living in total within the boundaries of the park.

Another limitation of this study, albeit anticipated and considered, was the lack of existing norms of data and/or a proper control group for the communities visited. With indigenous people in Amazonia, especially those in the restricted area of MNP, there are reasons to closely consider comparisons to matched groups from the surrounding regions. Using such comparison groups, especially those too far outside areas with Hg exposure may have introduced error associated with differences of nutrition, general health, education, and familiarity with formal testing. This is a familiar problem to researchers in the field [18,88] and the problem may demand multiple strategies from different research programs to achieve a clear understanding of the more subtle impacts associated with Hg. For the Matsigenka of MNP, we decided against using an outside control group and, instead, focused on the dose-related relationship of Hg and cognitive performance. This design may be the soundest approach and is not unlike the types of follow-up and secondary analyses that are found in benchmark studies of the field. For example, in the Faroe Island cohort studies, Grandjean et al. [88], created a control group from the children whose mothers had low mercury exposure. Similarly, Dos Santos-Lima et al. [19] used the top and bottom quartile of Hg levels in their participant sample for inferential analyses.

Outside of limitations, this study is bolstered by the theoretical framework provided by experimental cognitive research and offers a different approach to learning how Hg exposure may be impacting Amazonian communities. The tasks used for working memory were tailored to our sample and showed the relationships predicted by the Multicomponent Model and other literature on working memory. The findings encourage a closer look at how intelligence tests versus cognitive construct measures can be used in future assessments in isolated populations. More specifically, measuring working memory components may be a preferred index over IQ scores when there is sufficient cultural divergence between IQ batteries and participants. The results in this study suggest two options for detecting Hg impairment when working with isolated indigenous groups: the Corsi Block Test and the SOPT. The Corsi Block Test because it is more related to executive process than a simple storage task [48] and the SOPT because it is known to capture the unique executive abilities of strategic responding, internal organization and updating of information, and behavior regulation [51]. Moreover, the SOPT error scores correlate with tasks that are cornerstones of intelligence tests, such as the WAIS-III Block Design and Spatial Span subtests [89]. Both the Corsi Block Test and SOPT represent components of working memory that may be impaired by exposure to Hg and are also appropriate for use with indigenous people.

Future directions for this research can include more exploration of culturally modified cognitive tasks for Amazonian groups. Greater reference to the experimental literature on cognition can increase testing options for Hg studies when general intelligence tests and standardized comparison groups are not feasible in remote areas. Other areas of cognitive research should be explored for use in Hg studies including a deeper investigation into the types of executive functions sensitive to Hg exposure, such as updating, sequencing, and shifting of attention [34]. The research on executive functions beyond assessment of indigenous groups, a future direction for research can be to use current findings on intelligence for the development of intervention programs aimed at ameliorating or strengthening cognitive processes in ways that would be beneficial to Amazonia children and adults. Intervention programs can continue to be investigative while also addressing the immediate need for groups living among ASGM activity and other areas of Hg exposure.

## 5. Conclusions

The levels of methylmercury in hair of thirty participants from three different indigenous communities along the Manu River were found to be above the maximum threshold of 2.0 ppm [72,73] in all but three individuals, with levels exceeding the WHO limit by an average of 3.5×. The results point to widespread elevated Hg levels in even the most remote indigenous populations living in watersheds with illegal artisanal and small-scale gold mining—in this case between 300 and 400 km upstream from the nearest mine. This study also explored the association between methylmercury exposure and working memory using tasks chosen to measure the short-term storage and executive functions. Previous studies exploring the impact of Hg exposure have mostly relied on IQ tests or working memory sub-scales that are likely to be influenced by culture, education, and language and would not be appropriate for the Matsigenka living inside the restricted area of MNP [18,19]. Here, some tasks were modified by changing stimuli, instructions, and outcome variables to make the tasks more consistent with traditional Matsigenka culture. Results suggest that Hg exposure may impair cognitive processes that rely on executive functioning, and that these effects are seen at even relatively lower levels of Hg exposure and increase monotonically with increasing Hg concentrations in hair. The findings highlight the risk to Amazonian populations, especially those living in areas impacted by ASGM where mining activity increases levels of Hg in fish that are consumed as a main part of the diet [77,90].

## Figures and Tables

**Table 1:** Summary Statistics for Demographics, Health and Diet Indicators, and Cognitive Tasks.

Community		All Participants	Cacaotal	Maizal	Yomibato
n		30	11	3	16
Gender	F (M)	18 (12)	6 (05)	3 (0)	9 (7)
Age	average years (sd)	29 (13.8)	28 * (9.6)	45 (16.2)	26 (14.7)
Education					
none		8	3	2	4
primary		12	6	1	5
secondary		10	2	0	7
mean years		4.77 (3.8)	4.82 (3.68)	1.33 (2.31)	5.38 (3.99)
Fish Consumption					
Total per Week		4.10 (2.4)	4.58 (1.8)	3.73 (2.9)	3.98 (2.5)
Body Mass Index					
mean (sd)		22.93 (2.72)	22.8 (2.42)	24.38 (3.25)	22.75 (2.92)
<20		4	1	0	3
Anemia					
Hemoglobin		11.77 (1.42)	11.8 (1.56)	11.5 (1.65)	12.15 (1.25)
Males < 13.5, Females < 12		22	6	1	12
Hg (ppm)					
mean (sd)		7.05 (2.40)	6.04 (2.43)	11.49 (2.40)	3.61 (2.38)
min		1.81	2.84	9.67	1.81
max		14.21	11.42	14.21	11.43
Word Span					
mean accuracy (sd)		0.52 (0.19)	0.45 (0.19)	0.30 (0.11)	0.60 (0.16)
Corsi Block Span					
mean accuracy (sd)		0.59 (0.27)	0.47 (0.25)	0.25 (0.14)	0.73 (0.25)
SOPT Errors					
mean errors (sd)		4.23 (1.65)	4.72 (1.38)	4.67 (0.55)	3.81 (1.90)

* Three participants from Cacaotal did not know their age.

**Table 2:** Summary Statistics for Trail Making Test Sample Pilot Test.

Community		All Participants *	Yomibato	Cacaotal
n		19	14	5
Gender	F (M)	10 (9)	7 (7)	3 (2)
Age	mean years	23 (12.9)	22 (12.61)	21 (5.58)
Education				
mean years		6.63 (3.50)	6.29 (3.85)	7.6 (2.30)
Hg (ppm)				
mean (sd)		4.63 (2.92)	3.56 (2.54)	7.12 (2.64)
min		1.92	1.92	4.75
max		11.43	11.43	11.42
Trail Making Test A “Shades”				
mean percent accuracy (sd)		0.68 (0.35)	0.66 (0.34)	0.68 (0.42)
Trail Making Test B “Shapes & Shades”				
mean percent accuracy (sd)		0.31 (0.32)	0.34 0(.29)	0.08 (0.11)

* Maizal participants were not administered the TMT A & B.

**Table 3:** Correlation Matrix for All Variables across Communities.

	Age	Education	BMI	Hemoglobin	Fish Consumption	Hg	Word Span	Corsi Block	SOPT Errors
Age	1.00								
Education	−0.47 *	1.00							
BMI	0.26	0.29	1.00						
Hemoglobin	−0.01	0.04	0.16	1.00					
Fish Consumption	0.04	0.12	−0.14	−0.02	1.00				
Hg	0.50 *	−0.21	0.11	−0.13	−0.13	1.00			
Word Span	−0.56 *	0.63 *	−0.08	0.31	−0.13	−0.38 *	1.00		
Corsi Block	−0.44 *	0.59 *	−0.01	0.28	−0.04	−0.56 *	0.62 *	1.00	
SOPT Errors	0.10	−0.33	−0.20	−0.28	0.21	0.41 *	−0.34	−0.31	1.00

* *p* < 0.05.

**Table 4:** Multiple Linear Regression Models for Working Memory Components and Predictors.

*y*	Model Fit	*x*	*b*	95% CI	*p*
Word Span Accuracy	*R*^2^ = 0.50, Adj *R*^2^ = 0.44	Age	−0.00	(−0.01, 0.00)	0.16
	*F*(3,26) = 8.65, *p* < 0.01	Education	0.02	(0.01, 0.04)	0.01
		Hg	−0.01	(−0.03, 0.01)	0.35
Corsi Block Accuracy	*R*^2^ = 0.55, Adj *R*^2^ = 0.49	Age	0.00	(−0.01, 0.01)	0.83
	*F*(3,26) = 10.44, *p* < 0.01	Education	0.04	(0.01, 0.06)	0.00
		Hg	−0.04	(−0.06, −0.01)	0.01
SOPT Errors	*R*^2^ = 0.29, Adj *R*^2^ = 0.21	Age	−0.04	(−0.09, 0.01)	0.14
	*F*(3,26) = 3.57, *p* = 0.03	Education	−0.16	(−0.32, 0.00)	0.05
		Hg	0.23	(0.05, 0.42)	0.02

## Data Availability

The data presented in this study are available on request from the corresponding author. The data are not publicly available due to protection of participant privacy.

## Supplementary Materials

The following supporting information can be downloaded at: https://www.mdpi.com/article/10.3390/ijerph191710989/s1, Section S1: Word Span Stimuli in English, Spanish, and Matsigenka., Section S2: Analysis for Set Size Differences across Communities for Word Span, Corsi Block, and SOPT Errors.
