# Supplementary material for: Impairment in Working Memory and Executive Function Associated with Mercury Exposure in Indigenous Populations in Upper Amazonian Peru
Source: Int J Environ Res Public Health. 2022 Sep 2;19(17):10989. doi: 10.3390/ijerph191710989 (PMC9517927; doi:10.3390/ijerph191710989)
Supplement: Supplementary file 1 [file ijerph-19-10989-s001.zip › ijerph-1821111-supplementary.pdf]

Supplementary Section S1

Word Span Stimuli in English, Spanish, and Matsigenka

Category	English	Spanish	Matsigenka
Objects (12)	Arrow	Flecha	Chakopi
	Basket	Canasta	Kantiri
	Blanket	Manta	Magatsi
	Box	Caja	Tsivogo
	Clothes	Ropas	Manchakintsi
	Hook	Anzuela	Tsagarontsi
	Hut/House	Choza/Casa	Pankotzi
	Knife	Cuchillo	Kotsiro
	Pot	Olla de Metal	Hiromanka
	Rope	Soga	Tapetsa
Human (10)	Daughter	Hija	Shinto
	Ear	Oreja	Gempita
	Foot	Pie	Gititsi
	Hair	Pelo	Guisichi
	Hand	Mano	Ako
	Leg	Pierna	Boritsi
	Man	Hombre	Sürari
	Son	Hijo	Tomi
	Woman	Mujer	Tsinane
Nature (14)	Banana	Platano	Parianti
	Bird	Pajaro	Tsiriape
	Cassava	Yuca	Sekatsi
	Dog	Perro	Otsiti
	Firewood	Lena	Chichi
	Fish	Pez	Shima
	Flower	Flor	Kategari
	Moon	Luna	Kashiri
	Night	Noche	Chapini
	Plant	Pintura	Ana
	Red	Rojo	Kirahari
	River	Rio	Oakü
	Seed	Semilla	Okitsoki
	Sky	Cielo	Inküte
	Sun	Sol	Kenti
	Tree	Arbol	Inchanto
	Water	Agua	Nia

Supplementary Section S2

Analysis for Set Size Differences across Communities for Word Span, Corsi Block, and SOPT Errors

Word Span

Successful performance at the practice trials and lowest levels is important because it indicates that participants understood task expectations, could follow instructions, and could hold some verbal information in short-term memory. It was expected that recall would become more difficult with increased words presented and, indeed, mean recall accuracy for the set size of four items dropped to 0.76 (sd = 0.29) with perfect recall on at least one trial by 73% of participants. The mean recall accuracy dropped sharply for the set size of 5 items to 0.43 (sd = 0.25) with only 10% of participants getting a full score for a set size of 5 items. At the highest level, the mean accuracy was 0.24 (sd = 0.21) and only one participant could recall all 6 items. A repeated-measures ANOVA for set sizes was significant, F (3, 119) = 40.09 ($p < 0.01$), ω2 = 0.49, and Tukey's HSD post-hoc confirmed the largest differences between the set sizes between 4 and 5 ($p < 0.01$).

Corsi Block

At the lowest set size of 3, the mean accuracy was 0.86 (sd = 0.24) and 97% of participants were able to successfully recall block locations on at least one trial past successful completion of the practice trials. As expected, when set size increased the recall accuracy decreased: For the set size of four, the mean accuracy was 0.68 (sd = 0.32) and 67% of participants were able to recall at least one trial in perfect order. For the set size of five, the mean accuracy was 0.47 (sd = 0.32) with 40% of participants able to recall at least one trial in perfect order. For the highest set size of six, the mean accuracy was 0.49 (sd = 0.36) but the number of participants able to recall at least one trial in perfect order increased by 10% so that half could recall at least one trial correctly. The main effect of set size was significant, F (3,119) = 10.35, $p < 0.01$, ω2 = 0.19, and post-hoc comparisons showed differences between incremental set sizes of 4 and 5 (p = 0.04, Tukey's HSD).

SOPT Errors

At the lowest level, the mean error score was 0.47 (sd= 0.63) and 60% participants were able to complete the trial without any errors. For the set size of 6, the mean error score was 1.53 (sd = 0.82) with only 7% of participants able to respond without error. At the highest set size of 8 shapes, the mean error score was 2.33 (sd = 1.06) and all 30 participants made at least one error. An ANOVA for set sizes was significant, F (2, 89) = 46.93, $p < .01$, ω2 = 0.50, and a Tukey's post-hoc comparison showed significant differences between set sizes of 4 and 6 ($p < .01$) and set sizes 6 and 8 ($p < 0.01$).
